# Three factors underlying incorrect *in silico *predictions of essential metabolic genes

**DOI:** 10.1186/1752-0509-2-14

**Published:** 2008-02-04

**Authors:** Scott A Becker, Bernhard O Palsson

**Affiliations:** 1 Department of Bioengineering, University of California, San Diego, 9500 Gilman Drive 0412, La Jolla, CA 92093, USA

## Abstract

**Background:**

The indispensability of certain genes in an organism is important for studies of microorganism physiology, antibiotic targeting, and the engineering of minimal genomes. Time and resource intensive genome-wide experimental screens can be conducted to determine which genes are likely essential. For metabolic genes, a reconstructed metabolic network can be used to predict which genes are likely essential. The success rate of these predictions is less than desirable, especially with regard to comprehensively locating essential genes.

**Results:**

We show that genes that are falsely predicted to be non-essential (for growth) share three characteristics across multiple organisms and growth media. First, these genes are on average connected to fewer reactions in the network than correctly predicted essential genes, suggesting incomplete knowledge of the functions of these genes. Second, they are more likely to be blocked (their associated reactions are prohibited from carrying flux in the given condition) than other genes, implying incomplete knowledge of metabolism surrounding these genes. Third, they are connected to less overcoupled metabolites.

**Conclusion:**

The results presented herein indicate genes that cannot be correctly predicted as essential have commonalities in different organisms. These elucidated failure modes can be used to better understand the biology of individual organisms and to improve future predictions.

## Background

The dispensability and essentiality of genes in single-celled organisms is an extensively studied field [1] with multiple applications. Knowledge of which genes are indispensible is needed for the construction of minimal organisms, which are suggested as platforms for novel bacteria with beneficial characteristics [2]. For pathogenic organisms, lists of essential genes can be taken as lists of potential targets for new antibiotics [3]. In the field of metabolic engineering, non-essential gene deletions are used to create bacterial strains with better production characteristics [4].

Sizeable screens for essential genes have been undertaken in a number of organisms [1,5], necessitating significant time and resources. Alternatively, at least as far as metabolic genes are concerned, *in silico *methods can be used to predict gene essentiality. Such *in silico *studies have been undertaken for a variety of organisms, including *Escherichia coli *[6]*Saccharomyces cerevisiae *[5,7], *Helicobacter pylori *[8], *Staphylococcus aureus *[9], *Bacillus subtilis *[10], and *Mycobacterium tuberculosis *[11]. These methods are fast and require few resources. The rate-limiting step is the mandatory reconstruction of the metabolic network, which is a valuable resource to develop for a variety of other applications [12]. These reconstructions are currently available for a relatively small, but growing, number of microorganisms. For organisms without a reconstruction, methods to elucidate the context in which essential genes occur across many organisms have been described [1].

Multiple, simultaneous *in silico *gene deletions experiments have also been described; see for example [8,13]. In most organisms, any given individual metabolic gene is likely dispensable under most conditions, due to robustness properties that appear to be inherent to many biological networks [14,15]. Experiments in which multiple genes are removed from the organism are necessary to dig deeper into its capabilities. Of course, not all individually dispensable genes can be removed at once from an organism, meaning that a collection of single knock-out experiments cannot itself provide instructions for constructing a minimal organism. Double and higher simultaneous knock-out experiments can be technically challenging in the lab and complete coverage of the genome is virtually impossible due to the combinatorial explosion. As cited above, computational methods can easily predict the results of such higher knock-outs. While the computer time required for anything more than a comprehensive double-deletion study may be prohibitive, a many more knock-outs can be simulated *in silico *than can be performed *in vivo*. Computational studies can be used as screens to identify potentially interesting multiple knock-outs to pursue in the lab, as has been demonstrated for metabolic engineering applications [16,17].

Unfortunately, *in silico *methods for predicting gene essentiality are not perfect. There are four possible outcomes when comparing the results from *in silico *methods with experiments: true positives (TP), true negatives (TN), false positives (FP), and false negatives (FN). True positives occur when both the model and experiment indicate that a gene is essential, and true negatives occur when the model and experiment agree that a gene is nonessential. False positives occur when the model says a gene is essential, but experiments suggest otherwise. False negatives occur when the model says a gene is nonessential, but experiments indicate that it is essential. The overall success rate is given by the ratio of TP and TN to FP and FN. The best large-scale studies cite overall success rates in the vicinity of 90% [5,6,10], but nearly all cited success rates are inflated by the large number of non-essential genes that are correctly predicted. While these success rates are not inaccurate, the correct prediction of nonessential genes is less important than the correct prediction of essential genes. In false positive cases, one experiment, the deletion of that gene in the lab, can verify that a prediction is wrong. However, in false negative cases, only a comprehensive set of experiments (one attempted deletion per gene) can locate errors. When *in silico *studies are considered as screens for essential genes, perhaps for antibiotic target discovery, false-negative errors limit the usefulness of such screens. As detailed herein, when only experimentally-determined essential genes are considered for statistical purposes, success rates (or essential success rates) are lower.

There are several reasons for incorrect essentiality predictions, and incorrect predictions for a single organism are frequently studied and described in the publications that describe these predictions. Incorrect predictions are believed to usually occur for several reasons. False negative errors can be caused by incomplete definition of the biomass function, uncertainty in the growth medium used for experiments, and toxic-intermediate buildup. False positive errors can be caused by overly stringent definition of the biomass function, uncertainty in the growth medium, and the presence of unknown isozymes for a given reaction. The biomass function is central to the simulation of gene deletions, because a gene is predicted to be essential if its deletion results in the complete impairment of flux through this special reaction. The growth medium used for experiments is also very important because genes essentiality is dependent on what substrates are available for use. The buildup of toxic intermediates is difficult to simulate accurately with constraint-based methods because, in the absence of knowledge that the cell will produce a metabolite even if it cannot be broken down, there is no way to predict the production of toxic metabolites. The presence of unknown isozymes suggests that the organism is not understood as well as it could be.

While organism and gene specific explanations for incorrect predictions can be informative and lead to new discoveries, we have elected to study and classify incorrect predictions across organisms without trying to justify each inaccuracy by itself. Herein we report that genes that are incorrectly predicted as dispensable share common characteristics in multiple organisms. In terms of computational predictions, these genes are less connected in the network, more likely to be predicted inoperative, and connect to less overcoupled metabolites. Taken together, these characteristics suggest that incorrectly predicted genes are connected beyond the boundaries of known metabolism, both through limited knowledge of the reactions they catalyze directly and through the limited understanding of metabolism surrounding those reactions.

## Results and Discussion

### *in silico *vs. experimental gene deletions

We used six genome-scale metabolic networks [3,6-8,10,11] and a combined total of 13 experimental gene essentiality data sets [5,18-25]. These networks are all elemental and charge balanced, and they have been manually curated. In terms of included genes, these are the most complete networks for each organism that have been put together by hand and carefully validated.

**Figure 1:**
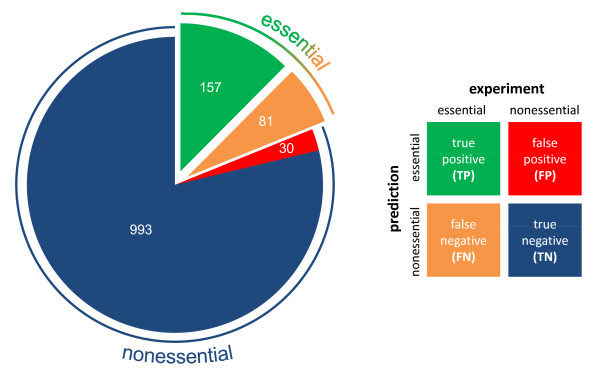
Essential genes in *E. coli*. This pie chart classifies all genes based on their essentiality on glucose minimal medium. The genes that are actually essential are in the green and orange regions, indicating correct and incorrect predictions, respectively. The genes that are not essential are in the blue and red regions, again indicating correct and incorrect predictions, respectively. The definitions for TP, FN, TN, FP are shown in a color-coded table for clarity.

Gene deletions were simulated using flux balance analysis, which is the most common method in use. In short, reactions that absolutely rely on a particular gene for catalysis were removed from the networks one at a time. If growth (biomass production) was still possible, the gene is predicted to be non-essential; otherwise, it is predicted to be essential. Full details are presented in the methods section. The gene essentiality experiments used herein vary in methodology and coverage; interested readers should consult the papers cited above for full details. It must be noted that the experimental gene essentiality results are almost certainly not perfect; for example, it is possible that a gene is refractory to the attempted knock-out methodology and yet is not absolutely essential for growth of the organism. Figure 1 compares computationally-predicted with experimentally-determined genes for *E. coli *on glucose minimal medium. The green region represents true positives (TP), where both prediction and experiment indicate a gene is essential. The orange region represents false negatives (FN), where a gene is predicted to be non-essential but in reality it is essential. The blue and red regions indicate genes that are not essential, and are predicted correctly and incorrectly, respectively. Although genes in the red region are also predicted incorrectly by the model, these mistakes are easily found with limited experimental screens. Genes in the orange region cannot be deciphered as incorrect predictions without a genome-wide experimental screen, because we cannot distinguish the orange region from the blue region without a full set of experiments. Reducing the number of FN genes is thus a worthwhile and important goal. Toward this end, we focus on the green (TP) and orange (FN) regions to elucidate the differences between the sets of genes of which they are comprised.

The basic characteristics of the networks and the experimental data sets are shown in Table 1. The final column lists the percentage of essential genes, as determined by the experimental studies, that the models correctly predict. This percentage is in all cases lower than the percentage cited in the papers detailing the reconstructions because we only consider experimentally-determined essential genes here. For statistical purposes, the papers describing the results of single-organism gene deletion studies also consider correctly predicted non-essential genes, which significantly outnumber the essential genes in most cases and generally are more often predicted correctly. As can be seen, if the *in silico *studies are used to identify potentially essential genes to test experimentally, even in the best cases, nearly a third of essential genes would be missed. These genes share certain characteristics that provide insight into cellular metabolism and the state of knowledge we currently have.

**Table 1:** The microorganisms and media conditions used in the study, together with the predicted gene essentiality results. The percentage of essential genes predicted correctly is a measure of how effective a screen the computational gene essentiality prediction really is.

**Organism**	**Total # of genes**	**Experimental media**	**# of TP genes**	**# of FN genes**	**% essential genes correct**
*E. coli*	1261	glucose MM	157	81	66.0
		glycerol MM	156	86	64.5
*S. cerevisiae*	750	glucose	63	95	39.9
		glucose (anaerobic)	47	109	30.1
		galactose	66	136	32.7
		glycerol	66	132	33.3
		ethanol	90	120	42.9
		rich	28	90	23.7
*H. pylori*	339	rich	36	39	48.0
*S. aureus*	619	rich (Forsyth et al.)	7	27	20.6
		rich (Ji et al.)	1	5	16.7
*M. tuberculosis*	661	Middlebrook	105	132	44.3
*B. subtilis*	844	rich	65	31	67.7

### Network Topology

Topological summary statistics (number of genes, number of reactions, number of gene associated reactions, number of metabolites) were noted for each metabolic network studied. These statistics were tightly correlated with each other; for example, a network with a larger number of genes is likely to have a larger number of reactions and metabolites (results not shown here). However, these statistics showed no significant correlation with the ability of a network to correctly predict the essentiality of genes. Model performance, at least in terms of predicting essential genes, does not appear to be related to model size. This lack of correlation suggests that the number of components (genes, reactions, etc.) in a network does not impact our ability to reconstruct an accurate network.

### Gene connectivity

A particular metabolic gene, either alone or in conjunction with other genes, encodes one or more enzymes responsible for one or more biochemical reactions. The associations between genes, enzymes, and reactions for each metabolic network we analyzed are publically available and are termed gene-protein-reaction associations (GPR's) [12]. Herein, we define the connectivity of a gene as the number of reactions it affects, as characterized by the GPR's. Depending on the organism, the mean connectivity for a gene is between one and three. The connectivity of a gene is a reflection of its understood prominence in the metabolic network, as measured by the number of discrete metabolic transformations it enables. Due to imperfect knowledge of the functions of genes, the connectivity of a gene is an estimate, and probably a low estimate. Because metabolic networks are reconstructed by only assigning functions to genes when they are relatively certain, the actual connectivity of a gene could be higher than the numbers given here.

**Figure 2:**
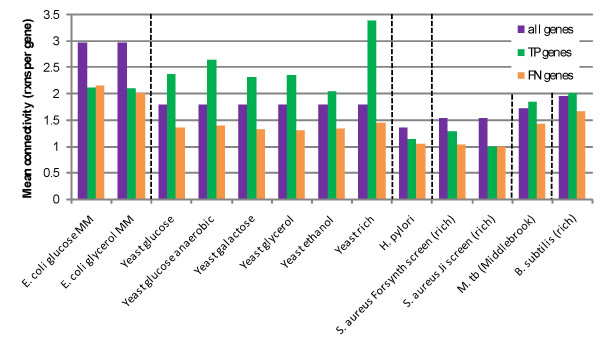
The mean connectivity of genes for each organism and media condition, plus the mean connectivity for TP and FN genes separately. On average, FN genes are less connected than TP genes, meaning that they are responsible for catalyzing fewer reactions.

The differences in gene connectivity across organisms/media conditions and between TP and FN genes are shown in Figure 2 and Table 2. With the notable exception of *E. coli *and one *S. aureus *dataset (which practically speaking, does not really have enough coverage to consider statistically), across the organisms, TP genes are more connected than are FN genes (p < 0.01). Within the network of each organism, an arbitrary but identical number of gene connectivities falling into the TP and FN class were randomly selected and their means compared. After repeating this procedure many times, *S. cerevisiae*, *H. pylori*, and *M. tuberculosis *all consistently had mean FN gene connectivities less than mean TP gene connectivities. *S. aureus *had mean FN gene connectivities less than mean TP gene connectivities approximately 94% of the time, and for *B. subtilis *it occurred approximately 83% of the time. As might be expected from Table 2, this trend does not hold in *E. coli*. This indicates that when all networks and experimental conditions are considered together, the trend is clear, but not all networks can be proven to have this trend with statistical significance.

**Table 2:** The connectivity of genes in the organisms studied. The overarching trend is that the mean connectivity of FN genes is less than the mean connectivity of TP genes for nearly all organisms and data sets.

**Organism**	**Overall Mean Connectivity**	**Experimental media**	**Mean connectivity of TP genes**	**Mean connectivity of FN genes**
*E. coli*	2.97	glucose MM	2.13	2.15
		glycerol MM	2.10	2.01
*S. cerevisiae*	1.80	glucose	2.38	1.36
		glucose (anaerobic)	2.64	1.40
		galactose	2.32	1.32
		glycerol	2.36	1.31
		ethanol	2.04	1.35
		rich	3.39	1.44
*H. pylori*	1.36	rich	1.14	1.05
*S. aureus*	1.54	rich (Forsyth et al.)	1.29	1.04
		rich (Ji et al.)	1.00	1.00
*M. tuberculosis*	1.72	Middlebrook	1.85	1.42
*B. subtilis*	1.96	rich	2.02	1.68

The outwardly obvious reason for this trend is that we do not have a comprehensive understanding of the function of FN genes. The lesser connectivity of FN genes suggests that they may be essential for reasons that are yet to be discovered or fully understood. The connectivity of essential genes may vary widely. However, we do not expect for it to fall into two groups corresponding to TP and FN unless the connectivity for FN genes is an artifact of an incomplete network *E. coli*, arguably the best understood microorganism, does not show this trend, supporting the notion that incomplete knowledge of gene function leads to the connectivity differences. We expect that as more is learned about the FN genes in other organisms their connectivity will increase and they will concurrently become TP genes as the reasons for their essentiality are understood.

### Flux variability and blocked genes

Given a metabolic network and an objective function, the allowable variability of the flux through each reaction can be computed with a series of linear programming problems [26]. In general, some fluxes can take a wide range of values (they have a wide flux span), some a smaller range, and some have no variability at all. Reactions that must not operate in a steady state are termed blocked reactions; they have no variability at all and are constrained by stoichiometry to carry zero flux. From a modeling standpoint, a reaction can be blocked for two reasons. First, the inputs and outputs determined by given environmental conditions (i.e. growth media) may not allow for a reaction to operate, but it would not be blocked under some different set of input and output constraints. This is called a condition-dependant gap. Second, the reaction may have one or more metabolites that are unavailable for production or consumption due to a network gap, which is basically a dead-end, or a condition-independent gap. This gap may be a modeling artifact due to incomplete knowledge of an organism, or a remnant that used to be functional in an ancestor of the organism. When gaps and blocked reactions occur in metabolic models, they are often viewed as an opportunity to discover something previously unknown about the organism [27].

To identify a relationship between gene essentiality and flux variability, we computed the maximum and minimum allowable flux through each reaction in each metabolic network, constraining the network to produce biomass at no less than 90% of the optimal rate. Because biomass production is permitted to take a range of values, as would be the case amongst any experimental population of cells, any reaction that has no flux span (meaning that its flux can only take a single value) must also be a blocked reaction. We found a widely variable number of blocked reactions in the networks, ranging from 75 in *H. pylori *to 888 in *E. coli *on glycerol minimal medium. We then mapped these reactions to genes, defining a gene as blocked if it is associated with at least one blocked reaction, and completely blocked if all reactions with which it is associated are blocked. Thus, a blocked gene may have some functionality in the network, but a completely blocked gene cannot.

**Figure 3:**
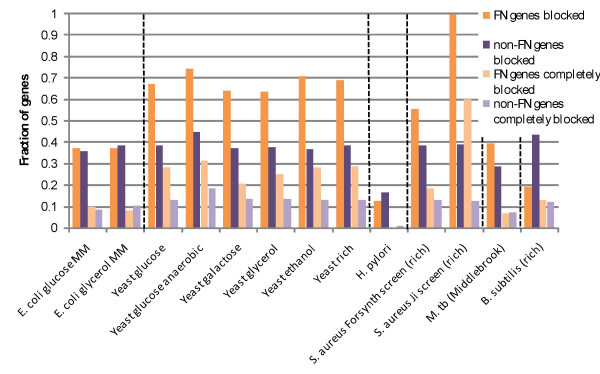
The fraction of FN and non-FN genes blocked and completely blocked. In most cases here, FN genes are more likely to be blocked (at least one of their reactions cannot be used) and completely blocked (none of their reactions can be used).

We found that when all organisms and media conditions are considered together, FN genes are more likely to be both blocked and completely blocked than non-FN genes (p < 0.02). However, this trend does not hold true on an individual level for each organism and experimental screen. The trend is largely driven by the highly uniform and significant results for *S. cerevisiae*, where all media conditions lead to the conclusion that with high certainty (p < 0.01), FN genes are more likely to be blocked and completely blocked than non-FN genes. *M. tuberculosis *(p < 0.01) and *S. aureus *(p < 0.06) also have reasonably compelling evidence for FN genes being preferentially blocked, but not completely blocked. The results are detailed in Figure 3 and Table 3.

**Table 3:** The fraction of blocked and completely blocked genes, both FN and non-FN.

**Organism**	**Media**	**Fraction of non-FN genes blocked**	**Fraction of FN genes blocked**	**Fraction of non-FN genes completely blocked**	**Fraction of FN genes completely blocked**
*E. coli*	glucose MM	0.36	0.37	0.09	0.10
	glycerol MM	0.38	0.37	0.10	0.08
*S. cerevisiae*	glucose	0.39	0.67	0.13	0.28
	glucose (anaerobic)	0.45	0.74	0.18	0.31
	galactose	0.37	0.64	0.14	0.21
	glycerol	0.38	0.64	0.14	0.25
	ethanol	0.37	0.71	0.13	0.28
	rich	0.39	0.69	0.13	0.29
*H. pylori*	rich	0.17	0.13	0.01	0
*S. aureus*	rich (Forsyth et al.)	0.39	0.56	0.13	0.19
	rich (Ji et al.)	0.39	1.00	0.13	0.6
*M. tuberculosis*	Middlebrook	0.29	0.40	0.07	0.07
*B. subtilis*	rich	0.43	0.19	0.12	0.17

Whereas the simplest explanation for the gene connectivity results above was incomplete knowledge about FN genes themselves, a better rationale for the blocked reactions here is incomplete knowledge of areas of metabolism closely associated with these genes. The network neighborhood of these genes is not completely understood. *E. coli *is again a very well-studied organism and it is not surprising that FN genes cannot be explained by incomplete knowledge of the surrounding network. *H. pylori *has a very compact metabolic network, with 45% fewer genes than the next smallest network. It also has one environment in which it is specialized, the human stomach. Thus, it is reasonable to conclude that this organism may have a reasonably comprehensively known metabolism. On the other hand, *S. cerevisiae *has a variety of factors complicating its metabolism, including the compartmentalization that is an essential feature of eukaryotic organisms. With metabolic processes spanning various organelles and intracellular transport mechanisms incompletely understood, it is logical that FN genes would result from a lack of knowledge of the surrounding metabolism.

### Overcoupled metabolite pairs

In genome-scale metabolic networks, certain pairs of metabolites occur in reactions together many times; for example, ATP and ADP. Some of these metabolite pairs can be classified as overcoupled based on statistical calculations that consider the individual connectivity of each metabolite and the network structure [28]. These overcoupled metabolite pairs are often associated with important cellular features such as energy transfer and charge balancing. Their functionality together is speculated to be important enough to have evolved beyond the point at which random connectivity would explain their co-occurrence. Even without knowing that these pairs of metabolites are overcoupled in a statistically significant manner, a casual observer would note that many of the pairs are highly important for cellular function.

We calculated overcoupled metabolites by the previously published method [28], using p < 0.01. We define a gene as associated with an overcoupled metabolite pair if it catalyzes at least one reaction in which at least one member of the overcoupled pair participates. The gene does not have to be associated with both members of the pair explicitly, but of course it is associated with both metabolites through the actions of whichever metabolite it directly influences. On average, 95% of genes in all models are associated with an overcoupled metabolite.

The overcoupling count for the i^th ^gene is calculated as

count = **p•Ŝ•G_i_**

where

**Ŝ **is the binary form of the stoichiometric matrix;

**G **is the gene-reaction association matrix (each row represents a reaction, each column a gene, and each binary entry indicates whether that gene is associated with that reaction); and

**p **is the overcoupled metabolite vector, with each entry specifying the number of overcoupling interactions with which a metabolite is associated.

This works out to the sum of the number of overcoupling interactions in which the compounds that are associated with a particular gene are involved, allowing compounds to be counted multiple times if they participate in multiple reactions. A simple example is presented in the methods section for clarity.

**Figure 4:**
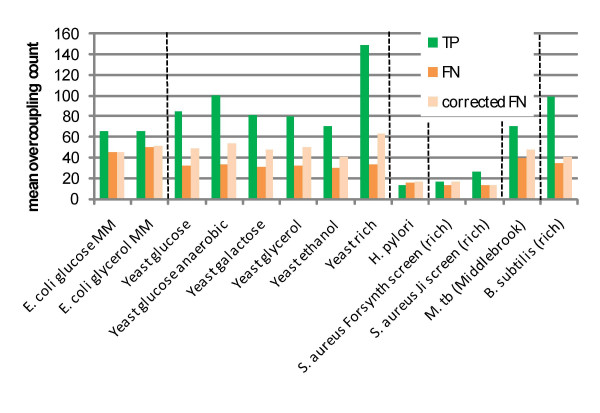
The mean overcoupling count for TP and FN genes, plus the FN count corrected for unequal connectivity of TP and FN genes. FN genes are on average connected to less overcoupled metabolites.

As previously shown (figure 2), FN genes are associated with fewer reactions on average than TP genes. Thus, we corrected for this bias by computing the overcoupling count for all gene-associated reactions multiplied by the fractional increased connectivity of TP genes. This correction factor is added to the overcoupling count for FN genes to get the corrected overcoupling count. The results are shown in figure 4 and table 4. In most cases, the FN genes, even after correction for unequal connectivity, are much less associated with overcoupled metabolites than the TP genes. Across all organisms, permutation testing gives p < 0.01. On an individual organisms basis, arbitrary numbers of genes were sampled from to provide some indication of the significance of the differences seen in figure 4. This procedure shows that most organisms have statistically significant differences between TP and FN genes (*E. coli *on glycerol and *H. pylori *are the key exceptions, and the small number of experimentally verified *S. aureus *essential genes stymies statistical tests).

**Table 4:** The mean overcoupling counts for each organism and media condition.

**Organism**	**Experimental media**	**TP mean overcoupling count**	**FN mean overcoupling count**	**Corrected FN mean overcoupling count**
*E. coli*	glucose MM	65.96	45.79	45.55
	glycerol MM	65.10	49.70	50.85
*S. cerevisiae*	glucose	84.87	32.05	48.55
	glucose (anaerobic)	99.74	33.93	53.19
	galactose	81.65	31.13	47.80
	glycerol	80.47	32.03	49.63
	ethanol	70.73	29.62	40.88
	rich	149.57	33.97	63.51
*H. pylori*	rich	13.58	15.62	16.50
*S. aureus*	rich (Forsyth et al.)	16.29	12.85	16.88
	rich (Ji et al.)	26.00	13.60	13.60
*M. tuberculosis*	Middlebrook	70.21	39.55	47.64
*B. subtilis*	rich	99.35	35.16	40.74

Because FN genes, on average, interact less with overcoupled metabolites, they are less likely to be tied into important, evolutionarily conserved metabolic processes, at least *in silico*. It is possible that the FN genes are responsible for reactions beyond what is currently known, similar to the proposed reason that FN genes have lower connectivity. It is also possible that the reactions with which FN genes are associated are not completely correct. For example, some of these reactions may have alternative substrate/product pairs that are highly important for the network.

## Conclusion

Herein we have demonstrated that incorrectly predicted essential metabolic genes have network level differences that are largely conserved across organisms. These differences are (1) a smaller mean number of reactions per gene, (2) a larger percentage of blocked genes, and (3) a smaller overcoupling count.

These three differences all rely on the interactions between networks components. Fundamentally, gene essentiality is a network-level property, so it is to be expected that explanations will rely on the network as a whole. We did not find any explanation for incorrect gene essentiality predictions based on simple statistics such as rudimentary network size metrics.

The results suggest that incomplete knowledge of the metabolic processes associated with essential genes and the immediately surrounding metabolic processes are driving forces in incorrect gene essentiality predictions. These factors in most cases cannot with statistical significance explain incorrect gene essentiality predictions in *E. coli*, the best characterized microorganism considered here. One might expect, based on the numbers for *E. coli *shown in Table 1, that roughly a third of FN genes cannot be described with these explanations. Thus, further study of this topic is warranted.

One potentially fruitful area may be a comparative analysis of more precise network roles of FN genes vs. those of TP genes. One could, for example, computationally predict the necessity of each gene in the network for a variety of functions other than growth, such as redox balance or energy production. This may allow the determination of imperfectly understood areas of metabolism, even in well studied organisms. We foresee increased comparative analysis of microbial metabolism as more networks become available, akin to the growth of genome sequence comparisons from a curiosity to the essential tool that is BLAST today.

## Methods

### Metabolic network setup and *in silico *gene deletions

Metabolic networks for all six organisms were obtained as SimPheny (Genomatica, San Diego, CA) output files and imported into the COBRA Toolbox [29] in Matlab (The Mathworks, Inc., Natick, MA) using the readCbModel command with the SimpheyPlus format. Media conditions were set by using exchange fluxes to allow inputs to the model that are consistent with each published experimental gene deletion study.

Gene deletions were simulated using the singleGeneDeletion command in the COBRA Toolbox. The set of zero or more reactions that cannot occur without the presence of each gene were removed from the model, and we attempted to simulate growth. If no growth was possible, the gene was predicted to be essential. The results from the *in silico *experiments were compared with previously published experimental results to distinguish TP genes from FN genes (and both from genes that are not essential experimentally). Each gene in each organism under each media condition was identified as TP, FN, or not essential.

### Gene connectivity

Given the boolean gene-protein-reaction associations, the COBRA Toolbox automatically produces a binary matrix **G **describing the associations between genes and reactions. The number of non-zero entries in each column describe the connectivity of a single gene. The mean connectivity of TP and FN genes was determined with simple arithmetic. All graphs were made in Excel (Microsoft, Redmond WA).

### Flux variability and blocked reactions

The flux variability of each reaction in each network under each set of media conditions was determined using the fluxVariability command in the COBRA Toolbox, constraining biomass production to be no less than 90% of maximum. Reactions that cannot take any flux are found this way and termed "blocked." These blocked reactions are mapped back to genes through **G**, and genes associated with only blocked reactions are termed completely blocked; those associated with one or more blocked reactions are termed blocked.

### Overcoupled metabolites and overcoupled count

Overcoupled metabolites are computed with the same procedure as has been previously published [28]. The metabolite coupling matrix **M **is calculated as

**M **= **Ŝ•Ŝ^T^**

where **Ŝ **is the binary form of **S**.

**M **is a symmetric matrix with off-diagonal elements indicating the number of reactions in which two metabolites (rows and columns of **M**) co-participate. The diagonal elements give the total number of reactions in which each metabolite appears.

Overcoupled metabolites are determined by redistributing the elements of **Ŝ **such that the diagonal elements of **M **remain the same but the off-diagonal elements vary, in effect simulating the effects of random co-occurrence of metabolites but maintaining the connectivity structure of the network. After many redistributions, p values can be determined by comparing the actual value of **M**_ij _to the random distribution of values. We used only metabolites that are overcoupled with p < 0.01.

The overcoupled count for each gene was calculated as described above. As an example, consider a gene that catalyzes two isomerization reactions:

A -> B

B -> C

A is a member of one overcoupled metabolite pair, B is a member of 3 overcoupled metabolite pairs, and C is not overcoupled with any other metabolite. The count is 1 + 3 + 3 = 7 (1 for A, 3 for B in the first reaction, and 3 for B in the second reaction).

### Statistical testing

Except for determining which metabolite pairs are overcoupled, the statistics of which are summarized above and fully covered in [28], two statistical procedures were used to find p values. Comparisons across multiple organisms, for example, whether gene connectivity is less for FN genes, were analyzed with permutation tests. The data points were randomly assigned to two groups many times and the number of times the actual difference of means was greater (or less) than the random difference of means was noted. This number was divided by the number of randomizations to get a p value. In no case was the number of randomizations less than 10,000.

Comparisons within a dataset, for example, whether FN genes in *S. cerevisiae *are less connected than TP genes, were assigned a confidence score by randomly picking the same number of genes from each group and comparing their means. The number of times that the sampled mean for FN genes is less than the sampled mean for TP genes divided by the number of random samplings gives a confidence score or p value. No fewer than 10,000 randomizations were used.

## Authors' contributions

SAB conceived of the study, carried out all calculations, and wrote the first version of the manuscript. BOP participated in the design of the study and helped write the manuscript. All authors read and approved the final manuscript.
